# Prospective Comparison of Plasma Biomarker and Traditional Risk Factor Profiles for Incident Isolated Atherosclerotic Disease and Incident Isolated Abdominal Aortic Aneurysm

**DOI:** 10.3389/fcvm.2021.818656

**Published:** 2022-01-12

**Authors:** Stefan Acosta, Shahab Fatemi, Olle Melander, Gunnar Engström, Anders Gottsäter

**Affiliations:** ^1^Department of Clinical Sciences, Lund University, Malmö, Sweden; ^2^Vascular Centre, Department of Cardiothoracic and Vascular Surgery, Malmö, Sweden; ^3^Department of Internal Medicine and Emergency Medicine, Malmö, Sweden

**Keywords:** atherosclerosis, abdominal aortic aneurysm, risk factors, plasma biomarkers, lipoprotein-associated phospholipase, body mass index, diabetes mellitus

## Abstract

**Background:** Traditional risk factors for atherosclerotic disease (AD) are well-known, of which some are relevant also for abdominal aortic aneurysms (AAA). The present study compares the importance of plasma biomarkers and traditional risk factor profiles for incident AD without concomitant AAA (isolated AD) and AAA without concomitant AD (isolated AAA) during long-term follow-up.

**Methods:** In the Malmö Diet and Cancer Study—cardiovascular cohort, 5,381 participants were free from atrial fibrillation or flutter, AD (coronary artery disease, atherothrombotic ischemic stroke, carotid artery disease, or peripheral artery disease), and AAA underwent blood sampling under standardized fasting conditions between 1991 and 1994. Cox proportional hazards regression analysis was used to calculate hazard ratios (HR) with 95% CIs.

**Results:** During a median follow-up of 23.1 years, 1,152 participants developed isolated AD, and 44 developed isolated AAA. Adjusted HR for lipoprotein-associated phospholipase A2 (mass) (HR 1.53, 95% CI 1.14–2.04 vs. HR 1.05, 95% CI.99–1.12) was higher for incident isolated AAA compared to incident isolated AD, respectively. Mid-regional pro-adrenomedullin (MR-proADM) was associated with incident isolated AD (HR 1.17, 95% CI 1.1–1.25) and incident isolated AAA (HR 1.47, 95% CI 1.15–1.88). MR-proADM was correlated (*r* = 0.32; *p* < 0.001) to body mass index (BMI), and BMI was associated with increased risk of incident isolated AAA (HR 1.43, 95% CI 1.02–2). No participant with diabetes mellitus (DM) at baseline developed isolated AAA (0/44), whereas DM was associated with an increased risk of isolated AD (HR 2.57, 95% CI 2.08–3.18). Adjusted HR for male sex (HR 4.8, 95% CI 2.42–9.48, vs. HR 1.76, 95% CI 1.56–1.98) and current smoking (HR 4.79, 95% CI 2.42–9.47 vs. HR 1.97, 95% CI 1.73–2.23) were higher in the incident isolated AAA group compared to the incident isolated AD group, respectively.

**Conclusions:** The data supports the view that components of vascular inflammation and cardiovascular stress drives AAA development, whereas glycated cross-links in abdominal aortic wall tissue may have a plausible role in reducing AAA risk in individuals with DM.

## Introduction

Atherosclerotic disease (AD) is the main leading cause of death in the world due to coronary and cerebral artery disease (1). Atherosclerotic peripheral artery disease is an important underlying cause of death (2), and the presence of chronic limb-threatening ischemia carries a high risk of lower extremity amputation, especially in individuals with diabetes mellitus (DM) (3). Abdominal aortic aneurysm (AAA) develops and grows slowly before potential rupture many years later but is undoubtedly an important cause of death as a rupture is often fatal (4). The traditional risk factors for AD are age, male sex, smoking, hypertension, DM, and high cholesterol (5). Although individuals with AAA share similar risk factors as those with AD, DM has in contrast repeatedly been shown to be a protective factor for the development of AAA (6–8).

While knowledge about the pathogenesis of atherosclerosis is substantial, it is less clear about mechanisms leading to AAA development (9). Plasma biomarkers play a critical role in the definition, prognosis, and decision-making in acute cardiovascular events. C-reactive protein is an established plasma biomarker for inflammation associated with cardiovascular disease (10). Several more novel biomarkers may reflect different aspects of the early development of cardiovascular disease (11). Lipoprotein-associated phospholipase A2 (Lp-PLA_2_) is an enzyme produced by macrophages and considered to be a biomarker for platelet activation with subsequent vascular inflammation (12). Increased levels of Lp-PLA_2_ have been associated with the early development of both peripheral arterial disease (13) and AAA (14). The hemodynamic plasma biomarker B-type natriuretic peptide has also been associated with both an early subclinical development of peripheral (15) and carotid (16) artery disease and AAA (17) decades before clinical manifestation in individuals free from disease. A biomarker of neurohormonal activation, mid-regional-pro-adrenomedullin (MR-proADM), has also been associated with the prediction of both carotid artery disease (16) and AAA (18). It appears that these biomarkers reflect the early onset of cardiovascular disease, reacting to either slow development of peripheral arterial atherosclerotic stenosis and/or abdominal aortic wall degeneration and aneurysm development. The above-mentioned plasma biomarkers have previously been analyzed in relation to incident cardiovascular disease in the same cohort (19), but to enhance the current understanding of the importance of plasma biomarker profiles for the development of either AD or AAA, it is of great interest to analyze plasma biomarker profiles in patients that either exclusively develops incident AD (isolated AD) or incident AAA (isolated AAA), extending the follow-up period for outcomes with 11 years resulting in a substantially increased number of both atherosclerotic and AAA events.

The primary aim of the present study was to evaluate inflammatory, hemodynamic, and neuro-hormonal plasma biomarkers and their association with incident isolated AD compared to incident isolated AAA during long follow-up of a large longitudinal, population-based prospective cohort study of middle-aged individuals.

## Materials and Methods

### Study Population and Data Collection

**Figure 1:**
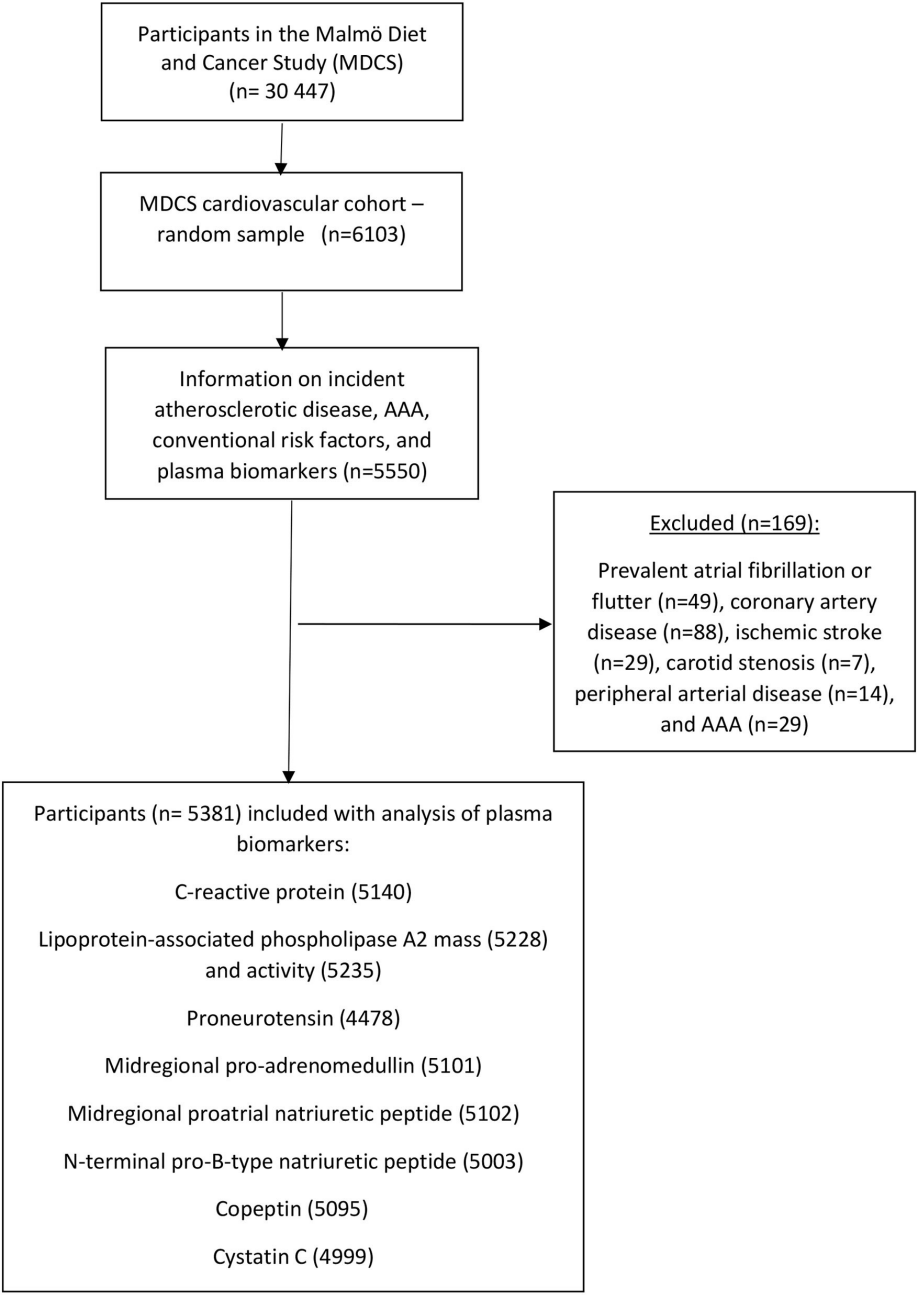
Descriptive flow diagram of study participants and plasma biomarker data. AAA, abdominal aortic aneurysm.

Men and women aged 46–73 years in Malmö, Sweden, were eligible to enter the Malmö Diet Cancer Study (MDCS) cohort, participated in baseline examinations between 1991 and 1996. The cohort was followed until December 31, 2016. Among 30,446 middle-aged individuals, a random subsample (*n* = 6,103) from this cohort was included in the MDCS cardiovascular cohort, of whom 5,550 individuals underwent blood sampling under standardized fasting conditions between November 1991 and February 1994 (18). The collection of fasting whole blood samples was performed at a median of 8.6 months after the baseline visit. The quality of biologically banked blood specimens based on biochemical analysis has been found to be optimal during at least up to 3.5 years of storage (20). Participants with prevalent AF, coronary artery disease, ischemic stroke, carotid artery disease, peripheral artery disease, or AAA were excluded, after which 5,381 remained to be included in the present study (Figure 1). The study was conducted in accordance with the World Medical Association Declaration of Helsinki and the protocol was approved by the Regional Ethical Review Board in Lund, Sweden (Dnr § LU 51-90, 2007/166). All subjects gave their written and oral informed consent for inclusion before study participation.

### Definitions

Current smoking was defined as self-reported regular smoking or smoking cessation within the last year. DM was defined as self-reported physician's diagnosis, use of anti-diabetic medication, or fasting venous blood glucose >6 mmol/L. Hypertension was defined as the use of antihypertensive medication or blood pressure ≥140/90 mmHg.

### Endpoint Ascertainment

The Swedish National Patient Register and the Cause of Death Register Participants were used to identify participants with a first registered diagnosis of AD via civic registration numbers. Diagnoses are coded using a Swedish revision of the International Classification of Disease (ICD), versions 8, 9, and 10. Incident AD was defined as a diagnosis of coronary artery disease, atherothrombotic ischemic stroke, carotid artery disease, or peripheral artery disease. Patients registered with AF prior to or simultaneously (±30 days) to ischemic stroke were labeled as AF-related ischemic stroke and excluded. AF-related ischemic strokes were followed up until the date of incident AF. AF was ascertained by ICD8-427.9, ICD9-427D, and ICD10-I48 codes. Follow-up was continued until the date of the first incident AD, death, or end of follow-up. The diagnoses of coronary artery disease, ischemic stroke, carotid artery disease, peripheral artery disease, and AAA in the Swedish National Patient register were separately validated by selecting a random sample of 100 study participants for each diagnosis (Supplementary Tables S1, S2: Validation of diagnosis of atherosclerotic cardiovascular disease and abdominal aortic aneurysm).

### Laboratory Measurements

Fasting total cholesterol and triglycerides were measured according to standard procedures at the Department of Clinical Chemistry, Skåne University Hospital Malmö. HbA1c was determined by ion-exchange chromatography, using the Swedish Mono-S standardization system; reference values were 3.9–5.3% in non-diabetic individuals.

Plasma biomarkers were measured from fasting plasma samples that had been frozen at −80°C immediately after collection (19). Storage time for the frozen plasma samples was ~13 years (19). CRP was measured by a high-sensitivity Tina-quant® latex assay (Roche Diagnostics, Basel, Switzerland). The average coefficient of variation (CV) was 4.59% (20). Lp-PLA_2_ was expressed as enzymatic activity and mass (quantity) (21). The lp-PLA_2_ activity was measured in duplicate using [3H]-platelet-activating factor as substrate. The range of detection was 8–150 nmol/min/ml, and the average CV was 5.78%. Lp-PLA_2_ mass measurements were performed using the commercially available second-generation PLAQ™ test (diaDexus Inc., South San Francisco, CA, USA) ELISA kit (22). The average CV was 4.62% on the 50 first participants in MDCS (22). Plasma-EDTA samples are stable for Lp-PLA_2_ activity and mass measurements within 7 days of collection for refrigerated samples and more than 10 years from the collection when stored at −70°C (22). Proneurotensin was measured using a chemiluminometric sandwich immunoassay to detect a proneurotensin fragment (23). Levels of MR-proADM were measured using immunoluminometric sandwich assays targeted against amino acids in the mid-regions of the peptide (BRAHMS AG, Henningsdorf, Germany) (24). The lower and upper limits of detection were 0.08 and 25 nmol/L, respectively. Mid-regional proatrial natriuretic peptide (MR-proANP) was measured using immunoluminometric sandwich assays targeted against amino acids in the mid-region of the peptide (BRAHMS, Berlin, Germany). NT pro-BNP was measured using the automated Dimension Vista Intelligent Lab System method (Siemens Diagnostics, Nürnberg, Germany) (19). Mean inter-assay CVs were ≤ 10% for MR-proADM, ≤ 10% for MR-pro-ANP, and 2.7% for NT pro-BNP. Copeptin was measured using a commercially available assay in the chemiluminescence/coated tube format (BRAHMS AG, Henningsdorf, Germany). Lower detection limit was 0.4 pmol/L and functional assay sensitivity (<20% interassay CV) was <1 pmol/L (25). Cystatin C was measured using a particle-enhanced immune-nephelometric assay (N Latex Cystatin, Siemens Diagnostics, Dade Behring, Deerfield, IL, USA) with a mean inter-assay CV of 4.3%.

### Statistical Analysis

Baseline characteristics were expressed as the median and interquartile range (IQR) for continuous variables and as total count and percentage for categorical variables. Comparison between nominal variables was performed with Fisher's exact test. Correlations between continuous variables were tested with the Pearson test. The proportional hazards assumption was tested by stratifying each categorical variable and the plots of the estimated log-log survival curves were found to be approximately in parallel and fulfilled the proportional hazards assumption. Cox proportional hazards regression analysis was used to calculate hazard ratios (HR) with 95% CIs to address confounding in baseline characteristics. The variables age, body mass index (BMI), total cholesterol, and plasma markers were tested for normal distribution with the Kolmogorov-Smirnov test and all these variables were log-transformed due to skewed distribution, and HRs were expressed per 1 SD increment. For statistical analyses IBM SPSS Statistics, version 26 (SPSS, Chicago, IL, USA) was used, and the level of statistical significance was *p* < 0.05.

## Results

### Participant Characteristics

The cumulative incidence of AD was 22.2% (1,196/5,381), in which 28.6% (622/2,178) for men and 17.9% (574/3,203) for women during a median follow- up of 23.1 years (IQR 16.3–24.2). Among the 1,196 patients, the first AD event was caused by coronary artery disease (*n* = 537; 44.9%), atherothrombotic ischemic stroke (*n* = 405; 33.9%), carotid artery disease (*n* = 89; 7.4%), and peripheral artery disease (*n* = 165; 13.8%). The cumulative incidence of AAA was 1.6% (88/5,381), and 68 (77.3%) of participant developing AAA were men. No participant with DM at baseline developed isolated AAA (0/44). DM at baseline was present in zero (0/44) and 8.4% (96/1,149) in participants developing isolated AAA and isolated AD, respectively (*p* = 0.044). Descriptive baseline risk factor characteristics for participants in the cohort without AD or AAA, with incident isolated AD, incident isolated AAA, and both incident AD and AAA, are shown in Table 1. Systolic blood pressure (*r* = 0.19; *p* < 0.001) and BMI (*r* = 0.32; *p* < 0.001) were correlated to MR-proADM.

**Table 1:** Descriptive baseline characteristics in participants with incident atherosclerotic disease (AD) and incident abdominal aortic aneurysm (AAA) during follow-up.

**Characteristic**	**No incident AD or AAA (*n* = 4,141)**	**Incident isolated AD (*n* = 1,152)**	**Incident isolated AAA (*n* = 44)**	**Incident AD and AAA (*n* = 44)**
Age years, median (IQR)	56.7 (51.8–62.0)	60.7 (55.8–64.3)	61.2 (54.3–63.4)	60.0 (53.7–62.3)
Male sex, %	1,525 (36.8)	622 (52.0)	31 (70.5)	37 (84.1)
Body mass index, kg/m^2^, median (IQR)	25.1 (22.9–27.7; *n* = 4,139)	25.6 (23.3–28.4; *n* = 1,149)	26.2 (24.4–28.5)	25.9 (24.6–27.6)
History of hypertension (%)	2,482/4,139 (60.0)	860/1,149 (74.8)	28 (63.6)	37 (84.1)
History of diabetes (%)	115/4,139 (2.8)	96/1,149 (8.4)	0/44 (0.0)	2/44 (4.5)
Current smoking (%)	1,077/4,139 (26.0)	398/1,149 (34.6)	24 (54.5)	31 (70.5)
Total cholesterol, mmol/L, median (IQR)	6.1 (5.4–6.8; *n* = 4,090)	6.3 (5.6–7.0; *n* = 1,138)	6.2 (5.3–7.0; *n* = 42)	6.2 (5.6–7.1; *n* = 39)
Triglycerides, mmol/L, median (IQR)	1.1 (0.8–1.5; *n* = 4,089)	1.3 (1.0–1.8; *n* = 1,137)	1.4 (1.0–2.1; *n* = 42)	1.6 (1.0–2.2; *n* = 40)
Hemoglobin A1c, %, median (IQR)	4.8 (4.5–5.1; *n* = 4,088)	4.9 (4.6–5.2; *n* = 1,134)	4.9 (4.7–5.1; *n* = 42)	5.0 (4.6–5.2; *n* = 40)
**Plasma biomarkers**, median (IQR)				
Lp-associated phospholipase A2 (activity, nmol/min/ml)	43.2 (35.5–51.9; *n* = 4,052)	46.6 (38.7–55.6; *n* = 1,101)	50.8 (40.7–63.0; *n* = 41)	54.1 (42.3–61.0)
Lp-associated phospholipase A2 (mass, ng/ml)	251.1 (212.0–312.3; *n* = 4,048)	269.0 (221.0–336.6; *n* = 1,098)	309.4 (262.0–375.6; *n* = 41)	278.5 (245.0–363.1; *n* = 41)
Copeptin (pmol/L)	5.0 (3.1–7.8; *n* = 3,928)	5.9 (3.5–9.4; *n* = 1,087)	6.3 (3.8–8.4; *n* = 39)	5.5 (2.9–10.0; *n* = 41)
Mid-regional proadrenomedullin (nmol/L)	0.44 (0.38–0.52; *n* = 3,934)	0.47 (0.40–0.55; *n* = 1,087)	0.50 (0.44–0.62: *n* = 39)	0.46 (0.39–0.56; *n* = 41)
Mid-regional proatrial natriuretic peptide (pmol/L)	65.4 (50.7–84.4; *n* = 3,935)	67.0 (50.9–88.5; *n* = 1,087)	57.5 (44.6–87.3: *n* = 39)	64.6 (46.6–87.0; *n* = 41)
N-terminal pro-B-type natriuretic peptide (pg/ml)	59.0 (33.6–106.0; *n* = 3,865)	65.8 (34.8–127.2; *n* = 1,062)	57.0 (27.0–100.8; *n* = 37)	69.0 (39.0–116.8; *n* = 39)
Cystatin C (mg/L)	0.75 (0.68–0.84; *n* = 3,872)	0.79 (0.72–0.89; *n* = 1,051)	0.81 (0.71–0.92; *n* = 37)	0.78 (0.70–0.87; *n* = 39)
Proneurotensin (pmol/L)	103.0 (75.5–146.8; *n* = 3,436)	107.4 (76.1–152.4; *n* = 971)	104.9 (81.6–160.2; *n* = 37)	119.7 (74.1–164.5; *n* = 34)
C-reactive protein (mg/L)	1.3 (0.6–2.6; *n* = 3,980)	1.6 (0.8–3.4; *n* = 1078)	1.6 (0.90–3.7; *n* = 41)	2.2 (0.75–3.2; *n* = 41)

*AD, atherosclerotic disease; AAA, abdominal aortic aneurysm; SD, standard deviation; IQR, interquartile range; Lp, lipoprotein*.

### Plasma Biomarkers for Incident Isolated AD and Incident Isolated AAA

Plasma biomarker levels at baseline in participants without AD or AAA, incident isolated AD, incident isolated AAA, and both incident AD and AAA, are shown in Table 1. The plasma biomarkers MR-proANP (*r* = 0.25 and *r* = 0.42), cystatin C (*r* = 0.23 and *r* = 0.36), and MR-proADM (*r* = 0.26 and *r* = 0.55) were significantly correlated to age at baseline in both participants with incident isolated AD and incident isolated AAA, respectively, whereas lipoprotein-associated phospholipase A2 (activity) (*r* = 0.059) and lipoprotein-associated phospholipase A2 (mass) (*r* = 0.09) levels were significantly correlated to age at baseline only in those with incident isolated AD. Lipoprotein-associated phospholipase A2 (activity) and MR-proADM were both associated with incident isolated AD and incident isolated AAA. NT pro-BNP, copeptin, cystatin C, proneurotensin, and C-reactive protein were associated with incident isolated AD, whereas lipoprotein-associated phospholipase A2 (mass) was associated with incident isolated AAA (Table 2). Adjusted HR for lipoprotein-associated phospholipase A2 (mass) (HR 1.53, 95% CI 1.14–2.04 vs. HR 1.05, 95% CI 0.99–1.12) was higher for incident isolated AAA compared to incident isolated AD, respectively.

**Table 2:** Adjusted hazard ratios (HR) for plasma biomarkers in relation to incident isolated AD and incident isolated AAA.

**Plasma biomarker**	**Incident AD (free from incident AAA)**		**Incident AAA (free from incident AD)**	
	HR^*^ (95% CI)	***p***	HR^*^ (95% CI)	***p***
Lipoprotein-associated phospholipase A2 (activity)	1.12 (1.04–1.19)	0.001	1.53 (1.11–2.11)	0.009
Lipoprotein-associated phospholipase A2 (mass)	1.05 (0.99–1.12)	0.096	1.53 (1.14–2.04)	0.004
Copeptin	1.09 (1.01–1.17)	0.018	0.98 (0.70–1.39)	0.92
Mid-regional proadrenomedullin	1.17 (1.10–1.25)	<0.001	1.47 (1.15–1.88)	0.002
Mid-regional proatrial natriuretic peptide	1.03 (0.97–1.11)	0.31	1.01 (0.71–1.43)	0.97
N-terminal pro-B-type natriuretic peptide	1.16 (1.08–1.24)	<0.001	1.13 (0.80–1.60)	0.49
Cystatin C	1.17 (1.11–1.23)	<0.001	1.13 (0.82–1.55)	0.47
Proneurotensin	1.07 (1.02–1.13)	0.010	1.09 (0.85–1.40)	0.49
C-reactive protein	1.17 (1.10–1.25)	<0.001	1.22 (0.88–1.68)	0.24

*AD, atherosclerotic disease; AAA, abdominal aortic aneurysm. Adjusted for age, sex, BMI, current smoking, hypertension and total cholesterol and each respective plasma biomarker*.

* *HR were expressed per 1 SD increment. Participants with incident AAA were excluded when assessing participants with incident AD and participants with incident AD were excluded when assessing participants with incident AAA*.

### Traditional Risk Factors for Incident Isolated AD and Incident Isolated AAA

Hypertension (HR 1.57, 95% CI 1.36–1.8), DM (HR 2.57, 95% CI 2.08–3.18), and total cholesterol (HR 1.10/SD increment, 95% CI 1.04–1.17) were associated with incident isolated AD in the adjusted Cox regression analysis, whereas BMI (HR 1.43/SD increment, 95% CI 1.02–2) was associated with incident isolated AAA (Table 3). Adjusted HR for male sex (HR 4.8, 95% CI 2.42–9.48, vs. HR 1.76, 95% CI 1.56–1.98) and current smoking (HR 4.79, 95% CI 2.42–9.47 vs. HR 1.97, 95% CI 1.73–2.23) were higher in the incident isolated AAA group compared to the incident isolated AD group, respectively. When exchanging hypertension for systolic blood pressure in the Cox regression model, systolic blood pressure was associated with incident isolated AD (HR 1.33/SD increment, 95% CI 1.25–1.42) but not associated with incident isolated AAA (HR 1.026/SD increment, 95% CI 0.73–1.44).

**Table 3:** Adjusted hazards ratios for traditional risk factors in relation to incident isolated AD and incident isolated AAA.

**Variables**	**Incident AD (free from incident AAA)**		**Incident AAA (free from incident AD)**	
	**HR (95% CI)**	***p***	**HR (95% CI)**	***p***
Age years, median (IQR)	1.66^a^ (1.55–1.78)	<0.001	1.82^a^ (1.30–2.54)	<0.001
Male sex, %	1.76 (1.56–1.98)	<0.001	4.80 (2.42–9.48)	<0.001
Body mass index, kg/m^2^, median (IQR)	1.04^a^ (0.98–1.11)	0.17	1.43^a^ (1.02–2.00)	0.038
History of hypertension (%)	1.57 (1.36–1.80)	<0.001	0.81 (0.42–1.56)	0.53
History of diabetes (%)	2.57 (2.08–3.18)	<0.001	b	b
Current smoking (%)	1.97 (1.73–2.23)	<0.001	4.79 (2.42–9.47)	<0.001
Total cholesterol, mmol/L, median (IQR)	1.10^a^ (1.04–1.17)	0.002	1.12^a^ (0.81–1.55)	0.51

*AD, atherosclerotic disease; AAA, abdominal aortic aneurysm. Adjusted for the variables in the table*.

a *HR were expressed per 1 SD increment. Participants with incident AAA were excluded when assessing participants with incident AD and participants with incident AD were excluded when assessing participants with incident AAA*.

^b^*No participant with DM developed AAA without concomitant AD so diabetes was excluded from the Cox regression model when analyzing independent risk factors associated with incident AAA*.

## Discussion

The present prospective cohort study showed that the plasma biomarker and traditional risk factor profiles for incident isolated AD and incident isolated AAA have many similarities but also several important differences. The plasma biomarkers lipoprotein-associated phospholipase A2 (activity) and MR-proADM were both associated with incident isolated AD and incident isolated AAA, whereas NT pro-BNP, copeptin, cystatin C, proneurotensin, and C-reactive protein were only possible to demonstrate an association with incident isolated AD. Interestingly, lipoprotein-associated phospholipase A2 (mass) was more elevated in those developing isolated AAA than in those developing isolated AD, suggesting that the development of AAA has a distinct component of vascular inflammation with subsequent athero-thrombotic disease.

The finding that MR-proADM (18) was found to be associated with incident isolated clinical significant AAA (Supplementary Table S2), implies on the other hand that AAA development may be driven by long-standing cardiovascular stress on the aortic wall. Systolic blood pressure at baseline was, however, not associated with incident isolated AAA. MR-proADM and BMI were associated with 47 and 43% increased risk of incident isolated AAA, respectively. Of note, the demonstrated association between MR-proADM and incident isolated AAA risk was adjusted for BMI. Elevated MR-proADM levels were highly correlated to BMI, and these individuals with higher BMI are perhaps more prone to future overweight or higher variability of BMI inducing MR-proADM production (26) and rendering them at higher risk for AAA development. A systematic review found, however, a statistically non-significant positive association between BMI and AAA presence (27). In contrast to the present study on participants developing AAA without concomitant AD, the inability to show an association between BMI and AAA might have been due to a series of patients with both AD and AAA in the AAA group (27) without the possibility to extract patients with AAA only.

In accordance with the finding that proneurotensin was associated with incident isolated AD, a previous study showed that proneurotensin was related to the risk of incident DM, cardiovascular disease, and cardiovascular mortality (23). The reason for elevated levels of this satiety hormone that appears to be associated with atherosclerosis is unclear, but neurotensin receptors are involved in cholesterol trafficking (28), and receptor-mediated neurotensin resistance has been suggested. Hence, the plasma biomarker profiles of incident isolated AD and incident isolated AAA does not support the view that both these diseases are inflammation-driven by a common pathway.

The most striking difference for the traditional risk factor profile was the increased number of participants with DM at baseline among those developing isolated AD compared to the total absence of participants with DM among those developing isolated AAA. DM has consistently been shown to be protective for both AAA development and growth (6–8). The hypothesized protective effects of DM on AAA include aortic wall changes such as decreased neoangiogenesis, inflammation, glycation, and cross-linking leading to increased resistance of collagen network to proteolysis, increased intima-media thickness, decreased wall stress, decreased protease expression and activity by matrix metalloproteinase, and plasmin and intraluminal thrombus formation (29). Hopefully, a better understanding of the mechanisms underlying the negative association between DM and AAA could help the development of innovative diagnostic and medical therapeutic approaches for the management of AAA.

In the original MDCS cohort, where none of the present plasma biomarkers were evaluated, 27,246 participants were included for the comparison of 3,020 individuals who developed coronary artery disease without concomitant AAA with 338 individuals who developed AAA without coronary artery disease (8): Male sex and current smoking were significantly stronger risk factors for the development of incident isolated AAA compared to incident isolated coronary artery disease, while systolic blood pressure and un-marital status were significantly stronger risk factors for incident isolated coronary artery disease compared to incident isolated AAA alone. Similarly, smoking has been found to be strongly associated with incident AAA and conferred a population-attributable risk of 47% (30). Male sex and current smoking were indeed significantly higher in those with incident isolated AAA compared to incident isolated AD in the present study.

The main limitation of the present study is the limited number of participants that developed isolated AAA. The risk of type 2 error when evaluating traditional and plasma biomarkers associated with incident isolated AAA was, therefore, substantial. Information about pack-years of smoking would have been useful to more accurately evaluate its impact on AD and AAA development. Repeated data on traditional risk factors and plasma biomarkers in the cohort at some point during follow-up would have been valuable to enable a more in-depth understanding of differences in pathogenesis between isolated AD and isolated AAA. Pre-analytical variation in biobanking procedures might also have influenced plasma biomarker outcomes. A quality control program for the storage of plasma in the Malmö Diet and Cancer Study has ensured that the long-term storage conditions at −80°C were optimal (20). In addition, the plasma samples used for biomarker measurement had not previously been thawed (31), assuring accurate plasma biomarker data for the present study. The very large prospective population-based cohort of middle-aged individuals at baseline, a median follow-up duration of 23.1 years, and validation of clinical endpoints were major study strengths. The validation showed that participants developing AD had an overwhelmingly symptomatic disease and that the AAA at diagnosis was large-sized and clinically relevant, which means that both groups had been exposed to harmful stimuli for a long time promoting their respective disease. The composite endpoint AD, composed of four clinical atherosclerotic manifestations, ensured meticulous scientific comparisons of risk markers between incident isolated AD and incident isolated AAA.

The plasma biomarkers lipoprotein-associated phospholipase A2 and MR-proADM were clearly associated with incident isolated large AAA in the adjusted Cox regression models, suggesting that elevation of these biomarkers indicates AAA susceptibility decades before clinical diagnosis of AAA. These biomarkers can, therefore, be of use in further research on AAA pathogenesis, screening for AAA, and potentially for the design of pharmacological drugs toward the growth of existing small AAA.

## Conclusion

The differences in plasma biomarkers and traditional risk factor profiles in participants developing isolated AAA compared to isolated AD in the present study may provide guidance for future research on AAA development. Data supports the view that components of vascular inflammation and cardiovascular stress drives AAA development, whereas glycated cross-links in abdominal aortic wall tissue may have a plausible role in reducing AAA risk in individuals with DM.

## Data Availability Statement

The raw data supporting the conclusions of this article will be made available by the authors, without undue reservation.

## Ethics Statement

The studies involving human participants were reviewed and approved by Regional Ethical Review Board in Lund, Sweden. The patients/participants provided their written informed consent to participate in this study.

## Author Contributions

SA was involved in the design of the study, data gathering, data analysis, and writing of the manuscript. SF was involved in the design of the study, data analysis, and critical review of the manuscript. OM, GE, and AG were involved in the design of the study and critical review of the manuscript. All authors contributed to the article and approved the submitted version.

## Funding

The Malmö Diet and Cancer study was made possible by grants from the Swedish Cancer Society, the Swedish Medical Research Council, the Swedish Dairy Association, the Albert Påhlsson and Gunnar Nilsson Foundations, and the Malmö city council. GE was supported by the Swedish Heart-Lung Foundation [2016-0315] and the Medical Faculty of Lund University. SA and AG were supported by grants from Research Funds at Skåne University Hospital, Region Skåne (430751), the Hulda Ahlmroth Foundation, and from the Swedish Government under the LUA/ALF agreement. The funding sources were not involved in study design, analysis, interpretation, writing, or submission of the manuscript.

## Conflict of Interest

The authors declare that the research was conducted in the absence of any commercial or financial relationships that could be construed as a potential conflict of interest.

## Publisher's Note

All claims expressed in this article are solely those of the authors and do not necessarily represent those of their affiliated organizations, or those of the publisher, the editors and the reviewers. Any product that may be evaluated in this article, or claim that may be made by its manufacturer, is not guaranteed or endorsed by the publisher.
