# Supplementary material for: Prospective Comparison of Plasma Biomarker and Traditional Risk Factor Profiles for Incident Isolated Atherosclerotic Disease and Incident Isolated Abdominal Aortic Aneurysm
Source: Front Cardiovasc Med. 2022 Jan 12;8:818656. doi: 10.3389/fcvm.2021.818656 (PMC8790118; doi:10.3389/fcvm.2021.818656)
Supplement: Supplementary file 1 [file Data_Sheet_1.PDF]

Table S1. Validation of diagnosis of atherosclerotic disease and abdominal aortic aneurysm

Validation of diagnosis of coronary artery disease

One hundred patients with diagnosis of coronary artery disease were randomly selected for the validation procedure using patient record data. Among 100 patients, 96 had acute myocardial infarction, of which 29 had ST-Elevation Myocardial Infarction (STEMI) and 67 had non-STEMI (NSTEMI). One patient died due to acute circulatory insufficiency of unknown cause but did not undergo autopsy. Three patients did not have acute myocardial infarction; one had transitory cerebral ischemic attack, one had acute pulmonary embolism, and one had acute biliary duct stone disease. Hence, acute myocardial infarction was confirmed in 96% of cases.

Validation of diagnosis of ischemic stroke

One hundred patients with diagnosis of ischemic stroke were randomly selected for the validation procedure using patient record data. Among 100 patients, 89 had stroke, whereof 87 of ischaemic origin. Two patients had intra-cerebral hemorrhage. It was unclear if one patient with fatal outcome had stroke or not, and autopsy was not undertaken. Of the ten patients that did not have stroke, four had transitory ischemic attack due to intra-cerebral thrombosis. Six patients did not have a cerebral ischemic event due to epilepsy (n=1), primary progressive aphasia (n=1), syncope (n=1), disorientation (n=1), headache (n=1) and acute lower limb ischemia (n=1). Among the 87 with ischemic stroke, the distribution of causes were the following: Intra-cerebral thrombosis (n=43; 49.4%), embolization secondary to atrial fibrillation (n=31; 35.6%), embolization due to carotid artery stenosis (n=7; 8.0%), carotid artery dissection (n=2), embolization secondary to endocarditis (n=1), unclear if symptomatic carotid artery stenosis or intra-cerebral thrombosis (n=2) and unclear if cardiac arrhythmias or intra-cerebral thrombosis (n=1). Among the 98 evaluable patients, 56 (57%) had an atherosclerotic cause of disease. The diagnosis of ischemic stroke was confirmed in 89% (87/98) of cases.

Validation of diagnosis of carotid artery disease diagnosis

One hundred patients with diagnosis of carotid artery disease were randomly selected for the validation procedure using patient record data. Among 100 patients, 57 had symptomatic (≥

60% stenosis of the internal carotid artery on color Doppler ultrasound) and 42 asymptomatic carotid artery disease. The proportion of operated patients with symptomatic and asymptomatic (≥ 70%) carotid artery disease were 85.9% (49/57), and 14.3% (6/42), respectively. One patient had coronary artery disease and was misdiagnosed. The diagnosis of carotid artery disease was therefore confirmed in 99% of the validation sample, and symptomatic carotid artery disease was confirmed in 57%.

Validation of diagnosis of peripheral artery disease

One hundred of patients with diagnosis of peripheral artery disease were randomly selected for the validation procedure using patient record data. Among 100 patients, 69 had critical limb ischemia, 13 had acute limb ischemia, 15 had intermittent claudication, and one had asymptomatic peripheral artery disease. Of the 13 patients with acute limb ischemia, 12 had acute thrombotic occlusion and one had an embolic occlusion. Two patients had venous insufficiency and were thus misdiagnosed. The diagnosis of peripheral artery disease could therefore be confirmed in 98% of cases and symptomatic peripheral artery disease in 97%.

Validation of AAA diagnosis

In all, 173 patients were diagnosed with intact abdominal aortic aneurysm (AAA) or ruptured abdominal aortic aneurysm (rAAA) between 1st Jan and 31st of December 2016. The proportion of individuals with ruptured and intact AAA were 18% and 82%, respectively. Therefore, eighty-two patients with AAA (I71.4) and 18 patients with rAAA (I71.3) were randomly selected for the validation procedure using patient record data. Two rAAA patients were simultaneously diagnosed with AAA and 98 patients remained for validation. Differences in characteristics between patients with AAA and rAAA are shown in Supplementary Table 2. The diagnosis of AAA or rAAA was confirmed in 94.9 % (93/98) of the patients. The five misdiagnoses were thoracic aortic aneurysm (n=1), common iliac aneurysm (n=1), multiple mycotic pseudoaneurysm in the abdominal aorta (n=1), chronic type B aortic dissection with secondary thoraco-abdominal aneurysm formation (n=1), and lower extremity artery stenosis (n=1).

Table S2. Validation of patients with AAA and rAAA

**Characteristics**	**AAA** (n=80)	**rAAA** (n=18)	P value
Confirmed diagnosis, (%)	76/80 (95%)	17/18 (94.4)	1.0
Median (IQR) age; years	76 (71 – 80)	84 (78 – 85)	<0.001
Male sex, (%)	58/76 (76.3)	12/17 (70.6)	0.62
Median maximal AAA diameter (IQR); mm	50 (40 – 64)	78 (56 – 98)	<0.001
Operated at the time of diagnosis, (%)	5/76 (6.6)	10/17 (58.8)	<0.001
EVAR	5	8	
Open repair	0	2	
Mortality within 30 days of diagnosis, (%)	0/76 (0)	11/17 (64.7)	<0.001
Initial mode of diagnosis, (%)			
Imaging due to AAA-related symptoms	2	16	
Accidental finding at any imaging	56	0	
Palpation	12	0	
Autopsy	0	1	
Organized ultrasound screening for AAA	2	0	
Non-organized ultrasound screening for AAA	4	0	

AAA; abdominal aortic aneurysm, rAAA; ruptured abdominal aortic aneurysm, EVAR; Endovascular aneurysm repair, IQR; interquartile range
